# Intraoperative optical coherence tomography imaging for assessment of anterior chamber gas fill

**DOI:** 10.3389/fopht.2024.1488764

**Published:** 2024-11-14

**Authors:** Michael Tseng, Avrey Thau, Carla Berkowitz, Abhijit Ramaprasad, Surendra Basti

**Affiliations:** Department of Ophthalmology, Northwestern Memorial Hospital, Chicago, IL, United States

**Keywords:** keratoplasty, optical coherence tomography, surgical technique, intraoperative imaging, intraocular pressure

## Abstract

**Introduction:**

During endothelial keratoplasty, anterior chamber gas is titrated to a desired fill, which is difficult to optimize by visualization alone. This study evaluates how an anterior chamber gas fill correlates with intraocular pressure (IOP) and iris-angle configuration as identified by optical coherence tomography (OCT).

**Methods:**

Eleven cadaveric eyes were studied in three configurations: baseline, air-fill just spanning limbus-to-limbus (“full-fill”), and air-fill maximally filling the anterior chamber (“overfill”). At each configuration, IOP was measured by Tonopen and iris-angle was determined by analyzing OCT images.

**Results:**

No differences in IOP or irisangles were identified between baseline and full-fill configurations (p=0.113 and p=0.152, respectively). When compared to overfill configuration, differences in IOP and iris-angles were identified for baseline (p<0.001 and p=0.001, respectively) and full-fill configuration (p=0.001 and p=0.039, respectively).

**Discussion:**

These findings highlight that en-face visualization of full-fill may not be indicative of IOP elevation. A significant difference in IOP and iris-angle exists between full-fill and overfill configurations. Intraoperative OCT can serve as a useful surrogate to identify the extent of fill.

## 1 Introduction

Anterior chamber (AC) gas fill is an essential step for achieving successful graft adhesion during endothelial keratoplasty (EK). During EK, the desired volume of gas injection can vary based on eye anatomy and surgeon preference – for instance, in eyes where no prior surgery has been performed a full anterior chamber air fill (as estimated by visualization of the bubble extending limbus to limbus) with minimal elevation of intraocular pressure (IOP) may suffice. However, in eyes with glaucoma filters, tube shunts, or previously failed penetrating keratoplasty, many surgeons believe that achieving a temporary overfill of the eye to achieve an elevated IOP for approximately 5-10 minutes is advantageous to improve graft adherence (1–3).

The optimal volume of the gas injected into the AC and the duration of time the AC is maintained with this volume during EK has not been definitively established in the literature. In this context, several intraoperative variables have been recognized to influence graft adherence. These variables include the initial gas volume and duration at that volume, subsequent gas volume and duration at that volume (if part of the initial gas was released), and the duration and timing of supine positioning (4–6). A frequently used technique, however, is to completely fill the anterior chamber to achieve an elevated IOP for at least some time before releasing part of the gas prior to discharging the patient (7). Since actual measurements of IOP intraoperatively brings in challenging elements related to sterility, it is not routinely used during surgery. Hence, most surgeons use the surgical or en-face view of the gas bubble to optimize its size. Using this approach alone however, can be inaccurate to determine if an adequate IOP has concurrently been achieved since IOP can vary with similar looking gas fills (8). To evaluate if reliable surrogates may be available to predictably estimate the extent of gas fill and IOP during its injection, we performed an ex-vivo investigation using cadaveric eyes and intraoperative anterior segment optical coherence tomography (iOCT). We examined anatomic changes in the iris and anterior chamber angle with varying volumes of air injection. Herein we report our study methods and findings.

## 2 Methods

This study was undertaken by evaluating cadaveric eyes that were harvested, preserved, and donated by Eversight (Chicago, Illinois, USA). Inclusion criteria included pseudophakic eyes that were harvested within 48-72 hours of death. Exclusion criteria included previous keratoplasty, phakic status, and eyes suitable for keratoplasty to a living patient.

**Figure 1:**
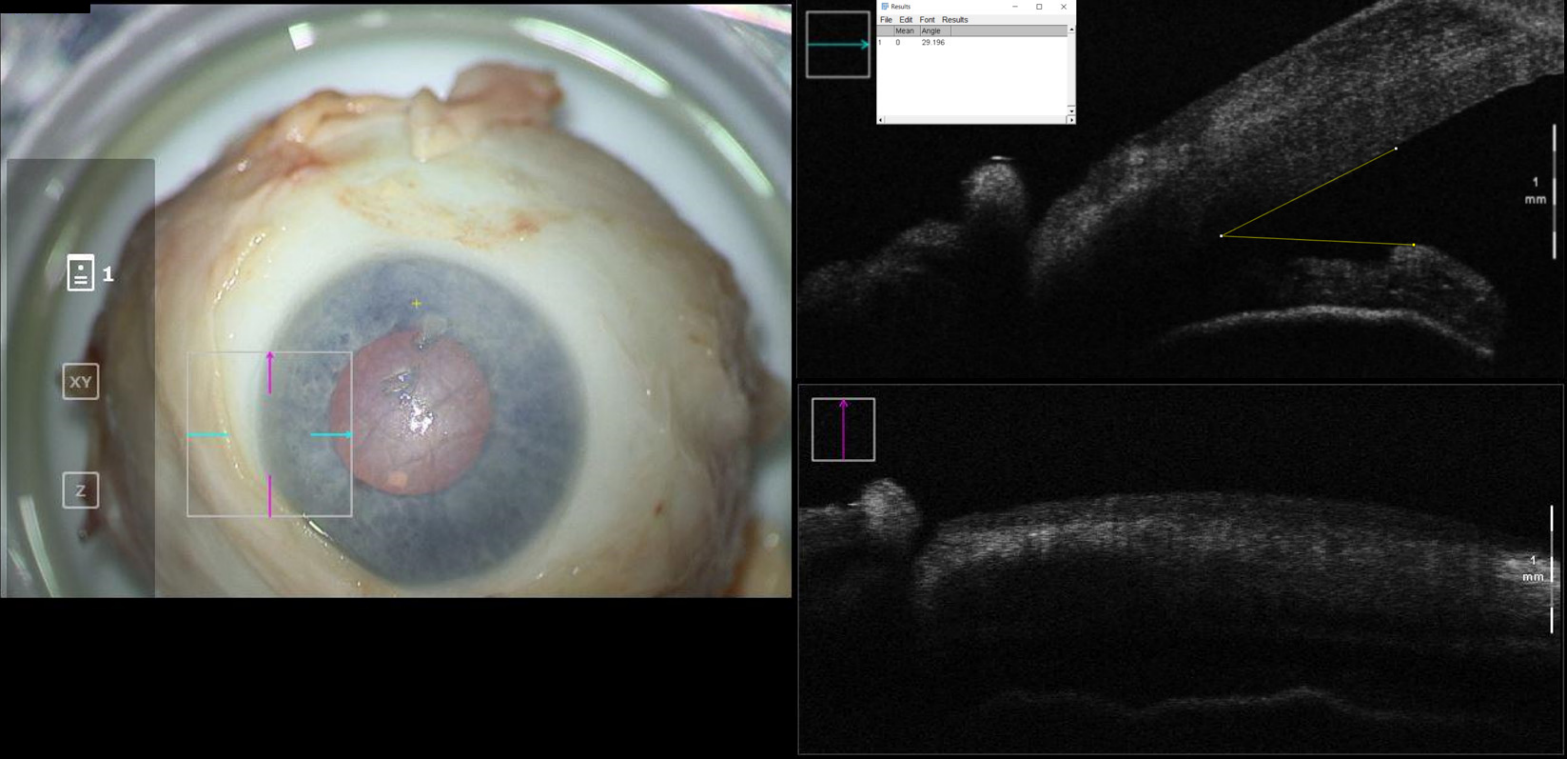
En-face view and intraoperative optical coherence tomography with marked iridocorneal angle measurements for a cadaveric eye at baseline configuration. Intraocular pressure in this eye was 21mmHg.

Study data were obtained at three different configurations: at baseline, with an air fill that just spanned limbus to limbus (“full-fill”), and an air fill that maximally filled the entire anterior chamber (“overfill”). At each configuration IOP and the iridocorneal angle were measured. The following method was used to achieve these study configurations and measurements:

The eye was first mounted under a surgical microscope with iOCT. IOP was palpated and if found to be too low, balanced salt saline (BSS) was injected into the vitreous cavity to emulate physiologic IOP. Next, IOP was measured by taking the average of three Tonopen (Reichert, Buffalo, New York) measurements and confirmed it was between 15-25 mm Hg. The iridocorneal angle was then imaged by anterior segment iOCT (Zeiss, Oberkochen, Germany) (**Figure 1**).

**Figure 2:**
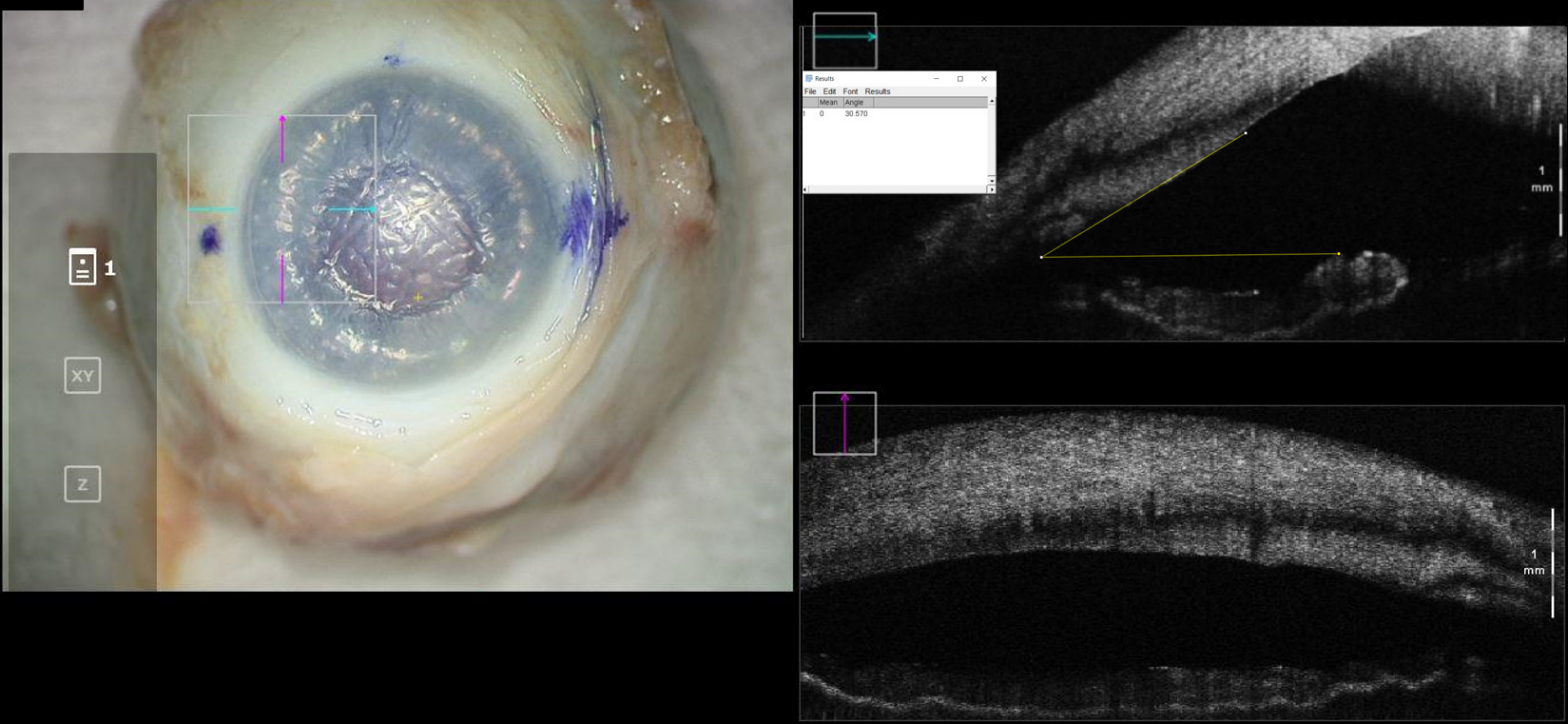
En-face view and intraoperative optical coherence tomography with marked iridocorneal angle measurements for a cadaveric eye at “full-fill” configuration. Intraocular pressure in this eye was 45mmHg.

**Figure 3:**
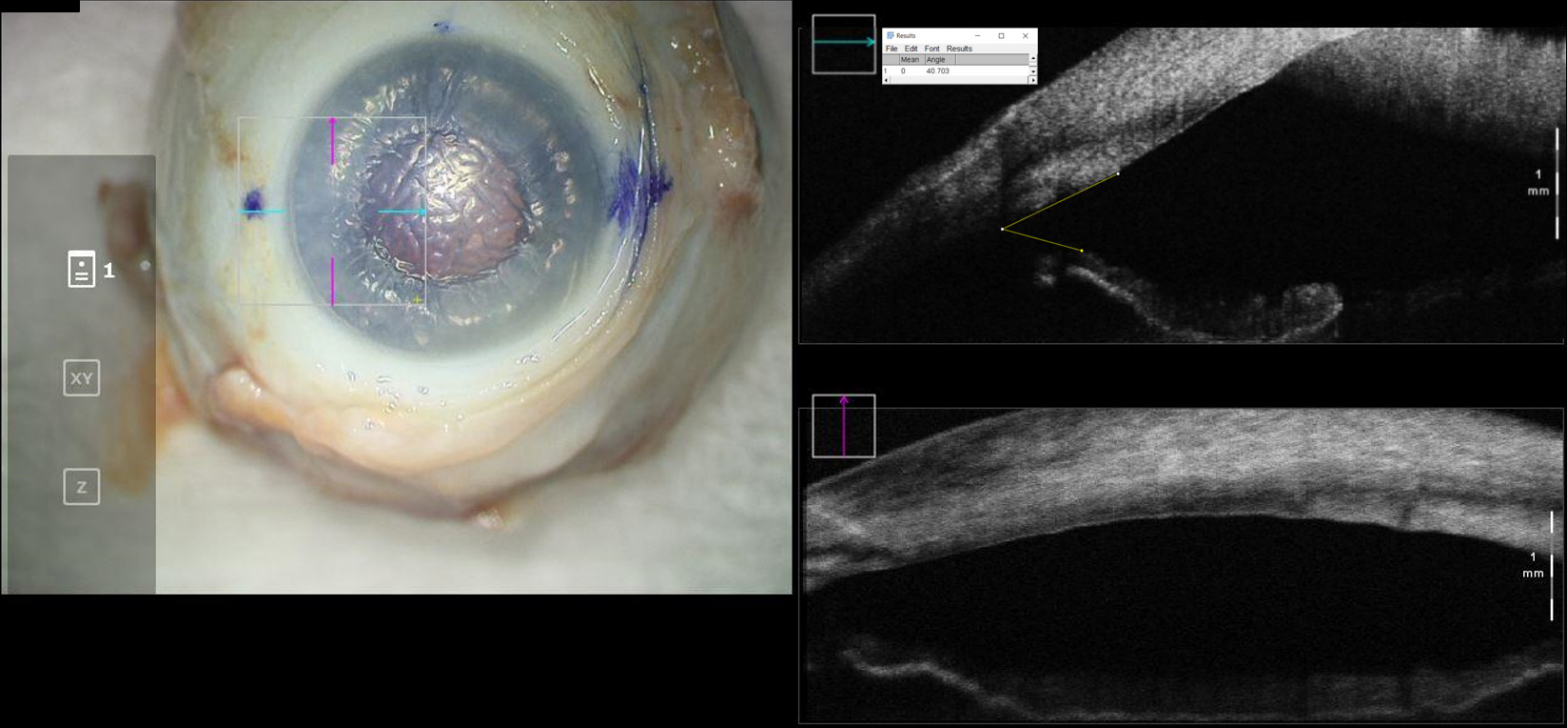
En-face view and intraoperative optical coherence tomography with marked iridocorneal angle measurements for a cadaveric eye at “overfill” configuration. Intraocular pressure in this eye was 99mmHg.

A 1-mm paracentesis was then created, and air was injected into the anterior chamber with a 30-gauge cannula to achieve a “full-fill,” which was considered an air injection to completely fill the anterior chamber with the air bubble just spanning limbus to limbus. The iridocorneal angle was again imaged with iOCT and IOP was measured (**Figure 2**). Finally, a 30-gauge needle was used to directly inject air into the anterior chamber through the cornea to achieve an “overfill,” which was air injection to maximally fill the anterior chamber and obtain an eye firm to palpation. Final anterior chamber iOCT images and IOP measurements were obtained (**Figure 3**). Care was taken to obtain iOCT images at the same location during all three of the above measurement timepoints.

The iOCT images were then exported, and two trained evaluators were blinded to analyze the OCT images. The iridocorneal angle was measured using ImageJ software. Collected data was analyzed using Microsoft Excel (Redmond, WA) and R statistical software (ver. 4.2.1; Vienna, Austria). The data means and standard deviations were summarized and compared with a paired t-test. A two-sided 5% significance level was used for all analysis.

## 3 Results

In total, 11 cadaveric eyes were included in the study. The mean IOP with standard deviation at baseline, full-fill, and overfill configurations were 22.7 ± 11.6, 23.7 ± 11.6, and 61.33 ± 24.4, respectively. No statistical difference was found between baseline and full-fill IOP (p = 0.113). When compared to the overfill configuration, differences in IOP were identified with both the baseline (p < 0.001) and full-fill (p = 0.001) configurations.

The mean measurement for iridocorneal angles with standard deviation at baseline, full-fill, and overfill configurations were 24.71 ± 10.2, 29.61 ± 5.8, and 35.82 ± 8.4 degrees, respectively. No statistical difference was found between baseline and full-fill iridocorneal angles (p = 0.152). When compared to the overfill configuration, differences in iridocorneal angles were identified with both the baseline (p = 0.001) and full-fill (p = 0.039) configurations.

## 4 Discussion

Graft detachment is the most common complication after endothelial keratoplasty (9). A wide variety of techniques have been described to improve graft adherence including scraping of the recipient stromal bed, mid stromal venting incisions, removing fluid from the graft/host interface with gentle corneal massage, and increasing the time of anterior chamber air fill (7, 10–12). There are studies that suggest a complete air fill and higher initial IOP are critical components to prevent graft detachment (7, 11, 13). It has been noted, however, that an anterior chamber completely filled with air can result in a wide range in IOP, even when the surgeon is aiming for a slightly elevated IOP (8, 11, 14, 15). During our study, we similarly noted difficulty in determining a difference in the appearance of the anterior chamber and iris configuration between the full-fill and overfill configurations under visualization from the surgical microscope alone. This may be due to how subtle the iris configuration change is while being on-axis with the surgeon’s view. Therefore, this study was undertaken to evaluate the relationship between iridocorneal angle identified by iOCT with IOP to help guide whether iOCT may be a useful surrogate to evaluate the extent of anterior chamber gas-fill.

This study provides insight into the use of iOCT during optimization of gas fill during EK. Notably, the IOP was consistently near physiologic when the iris was identified on iOCT to have minimal concavity and the IOP was consistently in the 50-60mmHg range when there was prominent concavity of the iris plane as identified by iOCT. The study data suggests that iris configuration on iOCT can serve as a quick, noninvasive, and reliable surrogate for estimating both extent of gas fill and IOP during endothelial keratoplasty. At the same time, surgeons should be aware that visualization of the refractile edge of the gas bubble being close to or at the iridocorneal angle (a sign used by many surgeons to suggest a full air fill) does not necessarily mean an elevated IOP. It is important to note that not all endothelial keratoplasties will require or benefit from an overfill of gas. Complications with air displacement into the vitreous cavity, zonular stress, or IOL dislodgement may occur in the presence of excessively high IOP or overfill. It is therefore important for surgeons to have the tools necessary to titrate both the fill and IOP.

Some studies have shown that longer air fill is correlated with better graft adherence, but there is no consensus on optimal volume and duration of air fill tamponade (13, 16–19). A study by Ćirković et al. showed that the size of the air bubble left at the end of DMEK surgery is critical for graft adhesion, with a significant improvement in graft adhesion of an 80% anterior chamber air fill compared to a 50% anterior chamber air fill (20). Graft dislocation has a strong association with subsequent graft failure. The rates of graft dislocation range from 0% to 43% for DSAEK and 4-95% for DMEK. This wide range of rates is a result of the various pre-operative, intraoperative, and postoperative factors that surround endothelial keratoplasty (12).

Intraoperative OCT has been shown to be a useful adjunct during EK. Studies have proven OCT to be useful in identifying Descemet’s membrane fragments, posterior stromal irregularities during Descemetorhexis, identifying graft orientation, graft apposition, reducing operative times, and aiding in physician decision making (21–23). None of these studies, however, have described specific iOCT markers to estimate gas fill in the context of EK. To our knowledge, ours is the first study to report this. We chose the iridocorneal angle as an investigational target of this study as it is often already in the view of the iOCT when used for many of the above-mentioned established benefits, such as routine assessment of the graft apposition. Another target that was considered is the anterior chamber depth. However, visualizing this amount of an anterior-posterior dimension at once requires changing the settings on the iOCT such that details of the posterior cornea may be lost.

Our study has limitations in that it is an ex-vivo study. Also, while the intent of the study was to aid in assessing gas fill during EK, donor insertion and subsequent iOCT of iris/angle was not performed. Our study only evaluated cadaveric, pseudophakic eyes with no prior surgeries. While our study included only pseudophakic eyes to standardize the lens-status variable amongst limited gifted tissues, it would be of interest for future studies to investigate the behavior in a phakic eye. Iris configuration is multifaceted and depends on multiple factors that were not evaluated in this study including scleral rigidity, vitreous status, axial lengths, small vs. large eyes, unicameral vs bicameral eyes, history of glaucoma surgery, pediatric eyes, etc. Intraoperative IOP similarly can vary based on vitreous status, posterior pressure, iris configuration, and history of glaucoma surgery. Our study also does not specifically address considerations to optimize duration of air fill to maximize graft adhesion.

In summary, our study demonstrates that visualizing iris orientation can serve as a quick and reliable surrogate for estimating IOP with air fill. A statistically significant widening of the iridocorneal angle and elevated IOP occur when an overfill is achieved. We believe that when available, utilizing iOCT during endothelial keratoplasty provides reliable cues to assist surgeons in optimizing desired air fill during EK.

## Data Availability

The raw data supporting the conclusions of this article will be made available by the authors, without undue reservation.
